# Genetic variants in the inflammation pathway as predictors of recurrence and progression in non-muscle invasive bladder cancer treated with Bacillus Calmette–Guérin

**DOI:** 10.18632/oncotarget.21222

**Published:** 2017-09-23

**Authors:** Stephen B. Williams, Ashish M. Kamat, Chinedu Mmeje, Yuanquing Ye, Maosheng Huang, David W. Chang, Colin P. Dinney, Xifeng Wu

**Affiliations:** ^1^ Department of Urology, The University of Texas MD Anderson Cancer Center, Houston, TX, USA; ^2^ Division of Urology, The University of Texas Medical Branch, Galveston, TX, USA; ^3^ Department of Epidemiology, The University of Texas MD Anderson Cancer Center, Houston, TX, USA

**Keywords:** polymorphisms, inflammation, bladder cancer, BCG, clinical outcome

## Abstract

Inflammation plays a critical role in the etiology of several cancers and may affect their clinical outcome. Our objective was to assess the association of genetic variants within the inflammation pathway with recurrence and progression among non-muscle invasive bladder cancer (NMIBC) patients with or without Bacillus Calmette–Guérin (BCG) treatment. We genotyped 372 single nucleotide polymorphisms (SNPs) in 27 selected genes within the inflammation pathway in 349 patients diagnosed with NMIBC, followed by internal validation in 322 additional patients. We used Cox proportional hazards regression analyses to identify SNPs as predictors for recurrence and progression. In the discovery phase, we identified 20 variants that were significantly associated with recurrence outcomes and 15 SNPs significantly associated with progression in patients treated with BCG but not in the transurethral resection (TUR)-only group. In BCG treated patients, rs7089861 was the only SNP significantly associated with risk of progression in both the discovery phase (Hazard Ratio [HR]=3.15, 95% Confidence Interval [CI]: 1.38-7.22, *P*<0.01) and validation phase (HR=3.84, 95% CI: 1.64-9.0, *P*=0.002; meta-analysis HR=3.47, 95% CI: 1.92-6.28, *P*<0.001). Two variants, rs1800686 and rs2071081, had probable association with HRs of the same trend in the discovery and validation groups (meta-analysis *P*=0.002). These findings supported the notion that genetic variation of inflammation pathway may impact clinical outcome of NMIBC patients treated with BCG immunotherapy. Further validation of these results in order to improve risk stratification to identify patients most likely to benefit from BCG treatment versus upfront radical cystectomy and future development of potential targeted therapies are warranted.

**Significance Statement:**

In a two-stage study, we identified several genetic variants in the inflammation pathway associated with recurrence and progression in early-stage bladder cancer. In particular, variant rs7089861 was validated for progression among patients who underwent BCG immunotherapy. Several other variants showed marginal association with recurrence or progression. These findings suggest that inflammatory pathway genetic variants may influence clinical outcome of bladder cancer patients and help to select patients most appropriate for BCG treatment.

## INTRODUCTION

There were an estimated 76,960 new cases and 16,390 deaths from bladder cancer in the United States in 2016 [1]. Non-muscle invasive bladder cancer (NMIBC) continues to be a challenge to treat. Although low-risk tumors can be appropriately treated with resection alone, most other tumors require adjunct intravesical therapies to improve clinical outcomes. While various chemotherapies have played significant roles in mitigating recurrences, Bacillus Calmette–Guérin (BCG) immunotherapy remains the standard first line intravesical agent for intermediate and high-risk NMIBCs.

After binding to urothelial cells via a fibronectin-dependent pathway, BCG is taken in by urothelial and inflammatory cells, which triggers a substantial inflammatory and immunologic response [2]. A unique characteristic of BCG-induced inflammation is the prominent early recruitment of polymorphonuclear leukocytes (PMNs), which are found in disproportionately large numbers (75% of immune cells) in the urine after BCG instillation [3]. As is typical of an inflammatory response to bacterial infections, the cytokine profile of IL-2, IL-12, and IFN-γ seen after BCG exposure is that of a Th1 response [4, 5]. Because local inflammation is a key mediator of BCG response, efforts have been made to improve BCG efficacy by amplifying the Th1 response after BCG-stimulation with the co-administration of various associated cytokines such as IFN-α2β, IL-2, IL18, and IFN-γ [4, 6]. Although preclinical studies have shown some benefit, these results unfortunately have not translated into the clinical setting [5]. Studies have been conducted in other malignancies to identify inflammation-related biomarkers, focusing mostly on circulating inflammatory molecules as well [7–9]. However, this approach has yet to identify reliable predictive biomarkers, mainly due to variation in methodology and sample processing. On the other hand, inherited genetic variations could be more reliable alternatives due to their stability and reproducibility [7].

There have been no studies to assess the association between genetic variants within the inflammatory gene pathway and outcomes in NMIBC. Moreover, there are no studies addressing potential pathway SNPs associated with recurrence and progression in NMIBC patients who received BCG immunotherapy. Further identification of unfavorable genotypes in the inflammatory gene pathway may select patients most likely to benefit from upfront radical cystectomy as well as patients’ likelihood of response to treatment. Thus, in the present study we utilize a pathway based approach to assess single nucleotide polymorphisms (SNPs) in the inflammatory gene pathway as predictors for recurrence and progression in NMIBC patients who underwent BCG immunotherapy and who underwent transurethral resection (TUR) alone.

## RESULTS

### Patient characteristics

The characteristics of the discovery and validation study populations are shown in Table 1 and Supplementary Table 1. A total of 671 (349 in discovery, 322 in validation) were included in the analysis. There was no significant difference between patients in the discovery and validation phases who received BCG versus TUR only according to age, gender, smoking status, tumor size and recurrence. In both the discovery and validation phases, patients who underwent BCG were more likely to harbor carcinoma *in situ* (42.4% v 14.6%, *P*<0.001) and have higher grade tumors (77.3% v 35.0%, *P*<0.001). Patients in the discovery phase were more likely to progress than patients who underwent TUR only, however, this was not significantly different in the validation phase. Conversely, focality was not significantly different among BCG and TUR patients in the discovery phase. However, there were significantly more multifocal tumors among BCG than TUR only patients in the validation phase (28.7% v 15.9%, *P*=0.004), respectively. Since BCG was primarily administered to those with higher risk of recurrence, we compared different BCG treatments on risks of recurrence and progression. Compared to iBCG, mBCG treatment was significantly associated with reduced risks of recurrence and progression in both the discovery and validation phases (*P*<0.01, data not shown).

**Table 1:** Demographics of the study populations according to treatment type

Variables^*^	Discovery			Validation		
	BCG (n=205)	TUR (n=144)	*P*-value	BCG (n=209)	TUR (n=113)	*P*-value
Age (y), Mean (SD) y	64.0 (10.2)	61.9 (12.7)	0.087	65.2 (10.9)	66.2 (10.7)	0.423
Sex, No (%)						
Male	170 (82.9)	111 (77.1)		177 (84.7)	96 (85.0)	
Female	35 (17.1)	33 (22.9)	0.175	32 (15.3)	17 (15.0)	0.949
Smoking status, No (%)						
Never	56 (27.3)	44 (30.6)		59 (28.2)	34 (30.1)	
Former	107 (52.2)	67 (46.5)		116 (55.5)	55 (48.7)	
Current	42 (20.5)	33 (22.9)	0.581	33 (15.8)	23 (20.4)	0.614
Carcinoma *in situ*, No (%)						
Yes	87 (42.4)	21 (14.6)		92 (44.0)	22 (19.5)	
No	111 (54.1)	118 (81.9)	**<0.001**	89 (42.6)	71 (62.8)	**<0.001**
Tumor size, No (%)						
1-2cm	27 (13.2)	19 (13.2)		31 (14.8)	17 (15.0)	
2-5cm	34 (16.6)	31 (21.5)		39 (18.7)	26 (23.0)	
>5cm	16 (7.8)	4 (2.8)	0.183	14 (6.7)	6 (5.3)	0.791
Stage						
Tis	18 (8.8)	2 (1.4)		70 (33.5)	68 (60.2)	
Ta	65 (31.9)	96 (67.1)		13 (6.2)	3 (2.7)	
T1	121 (59.3)	45 (31.5)	**<0.001**	121 (57.9)	35 (31.0)	**<0.001**
Grade						
G1	4 (2.1)	8 (5.7)		1 (0.5)	10 (8.8)	
G2	40 (20.6)	83 (59.3)		30 (14.5)	56 (49.6)	
G3	150 (77.3)	49 (35.0)	**<0.001**	168 (81.2)	42 (37.2)	**<0.001**
Focality						
1	70 (34.1)	64 (44.4)		47 (22.5)	45 (39.8)	
2	17 (8.3)	7 (4.9)		24 (11.5)	13 (11.5)	
Multiple	54 (26.3)	25 (17.4)	0.075	60 (28.7)	18 (15.9)	**0.004**
Treatment						
iBCG	121 (59.0)			105 (50.2)		
iBCG+mBCG	84 (41.0)			104 (49.8)		
Recurrence						
No	82 (40.0)	45 (31.3)		126 (60.3)	65 (57.5)	
Yes	123 (60.0)	99 (68.8)	0.094	83 (39.7)	48 (42.5)	0.630
Progression						
No	148 (72.2)	125 (86.8)		143 (68.4)	88 (77.9)	
Yes	57 (27.8)	19 (13.2)	**0.001**	66 (31.6)	25 (22.1)	0.720

SD: standard deviation; BCG: bacillus Calmette–Guerin immunotherapy; iBCG: induction BCG; mBCG: maintenance BCG; TUR: transurethral resection. Bold font denotes significant *P* values.

^*^Numbers for each variable may not add to total due to missing data.

### Associations between significantly opposing inflammatory genetic variants and recurrence

For this study we genotyped 372 tagSNPs in 27 candidate genes of the inflammation pathway (Supplementary Table 2) and analyzed their association with NMIBC outcome. In the discovery phase, we identified 20 SNPs that were significantly associated with recurrence in patients treated with BCG but were not significant in the TUR only group, and the hazard ratios (HRs) were in opposite direction (Table 2). Of these, rs3138056 in *NFKBIA* was the only SNP to have significant effect after correcting for multiple comparisons by Q value at a false discovery rate of ≤10% and conferred a 3.3-fold (95% CI, 1.83-5.8) increased risk of recurrence in patients who underwent BCG rather than TUR only treatment. To internally validate these results, we performed random bootstrap resampling of the significant SNPs for 100 iterations and listed the number of times that the *P* value was <0.05. In BCG-treated patients, three SNPs had highly consistent results, with bootstrap *P* values <0.05 for greater than 90% of samplings (rs3138056 in *NFKBIA*; rs951193 in *IL1R1* and rs4812997 in *CD40*) (data not shown).

**Table 2:** Significantly opposing inflammatory gene variants associated with recurrence in NMIBC patients in the discovery cohort who received BCG and TUR only treatment

SNP	Gene	MAF	MOI	TUR only				BCG			
				^#^ of events	^#^ of no events	HR (95% CI)^*^	*P*-value	# of events	# of no events	HR (95% CI)^*^	*P*-value
**rs3138056**	NFKBIA	0.32	REC	47\40\12	25\13\7	0.68 (0.36-1.28)	0.23	51\57\15	40\39\3	3.26 (1.83-5.8)	**6.20x10**^-5^
rs3732131	IL1R1	0.07	DOM	90\9\0	36\9\0	0.78 (0.38-1.63)	0.52	106\17\0	78\4\0	2.45 (1.36-4.4)	2.89x10^-3^
rs11607862	CD44	0.29	DOM	52\38\9	20\23\2	0.76 (0.5-1.16)	0.20	60\47\15	46\29\7	1.66 (1.13-2.44)	0.01
rs8193	CD44	0.33	DOM	44\44\11	20\21\4	0.91 (0.6-1.39)	0.67	54\53\15	40\33\9	1.65 (1.11-2.46)	0.01
rs228934	IL2RB	0.15	DOM	71\24\4	33\10\2	1.1 (0.69-1.75)	0.70	98\22\3	56\23\3	0.55 (0.34-0.88)	0.01
rs4755391	CD44	0.23	DOM	49\44\6	28\12\5	1.19 (0.78-1.81)	0.42	74\44\3	43\35\3	0.63 (0.42-0.94)	0.02
rs951193	IL1R1	0.05	DOM	88\11\0	38\6\1	0.75 (0.36-1.57)	0.45	110\12\1	78\4\0	2.03 (1.09-3.75)	0.02
rs7956804^#^	CD4	0.22	DOM	61\36\2	27\18\0	0.87 (0.56-1.34)	0.53	68\51\4	53\23\6	1.56 (1.05-2.32)	0.03
rs5743700	TLR2	0.06	DOM	88\11\0	40\4\1	1.09 (0.54-2.22)	0.80	114\7\1	71\11\0	0.39 (0.17-0.91)	0.03
rs2291473	ICAM1	0.15	DOM	69\28\2	35\9\1	1.34 (0.85-2.1)	0.20	91\32\0	53\27\2	0.62 (0.4-0.95)	0.03
rs4812997	CD40	0.30	REC	50\41\8	28\14\3	0.81 (0.37-1.79)	0.60	55\52\16	37\39\6	1.86 (1.05-3.29)	0.03
rs2241704	NFKBIB	0.23	DOM	48\44\7	27\17\1	1.3 (0.83-2.04)	0.25	84\34\5	45\33\4	0.63 (0.42-0.97)	0.03
rs17009223	CD34	0.08	DOM	82\16\1	36\8\1	0.86 (0.49-1.49)	0.58	102\20\1	76\6\0	1.74 (1.04-2.91)	0.03
rs2855537^#^	CD4	0.22	DOM	62\35\2	27\18\0	0.91 (0.59-1.41)	0.68	68\49\4	51\23\6	1.53 (1.02-2.27)	0.04
rs228935^#^	IL2RB	0.16	DOM	69\26\4	32\11\2	1.04 (0.65-1.64)	0.88	95\24\4	55\23\4	0.62 (0.39-0.98)	0.04
rs10911905	PTGS2	0.13	DOM	76\22\1	31\13\1	0.86 90.51-1.45)	0.57	88\32\3	67\14\1	1.57 (1.02-2.41)	0.04
rs709592^#^	CSF3	0.37	REC	40\43\16	20\21\4	1.36 (0.72-2.55)	0.34	49\47\25	33\43\6	1.64 (1.01-2.67)	0.04
rs4795418^#^	CSF3	0.37	REC	41\42\16	20\21\4	1.36 (0.72-2.55)	0.34	49\47\27	33\44\5	1.62 (1.01-2.59)	0.05
rs4794823^#^	CSF3	0.37	REC	41\41\16	20\21\4	1.36 (0.72-2.55)	0.34	49\47\27	33\44\5	1.62 (1.01-2.59)	0.05
rs709591^#^	CSF3	0.37	REC	40\43\16	20\21\4	1.36 (0.72-2.55)	0.34	49\47\26	33\43\6	1.62 (1.01-2.6)	0.05

^*^Adjusted by age, gender, smoking status, carcinoma *in situ*, tumor size, grade, and treatment.

SNP that continued to be significant by Q value after correcting for multiple comparisons with a false discovery rate of ≤ 10% is in boldface. ^#^SNPs with high linkage disequilibrium (LD; r^2^>0.8): rs7956804 and rs2855537 in LD; rs709592 and rs4795418 in LD; rs709592 and rs4794823 in LD; rs709592 and rs709591 in LD; rs4795418 and rs4794823 in LD; rs4795418 and rs709591 in LD; rs4794823 and rs709591 in LD.

TUR: transurethral resection; BCG: Bacillus Calmette–Guérin immunotherapy; MAF, minor allele frequency; MOI: model of inheritance; Rec: recessive; dom: dominant; HR: hazard ratio; CI: confidence interval.

Among the 20 significantly opposing SNPs identified in the discovery phase, we selected 5 SNPs for further validation based on whole genome genotyping results from previously unpublished data. These variants showed consistent association in the new validation group (Table 3). Among them, 2 SNPs (rs3732131 in *IL1R1* and rs228934 *in IL2RB*) remained significant in the meta-analysis, however, none reached significance during the validation phase.

**Table 3:** Results for the significantly opposing inflammatory gene variants in NMIBC patients with BCG treatment associated with recurrence in the discovery, validation and meta-analysis

*Recurrence*	Gene	MAF	MOI	Discovery		Validation		Meta-analysis	
SNP				HR (95% CI)^*^	*P*-value	HR (95% CI)^*^	*P*-value	HR (95% CI)	*P*-value
rs3732131	IL1R1	0.07	dom	2.45 (1.36-4.4)	**0.003**	1.04 (0.56-1.92)	0.903	1.63 (1.07-2.49)	**0.024**
rs228934	IL2RB	0.15	dom	0.55 (0.34-0.88)	**0.01**	0.85 (0.55-1.30)	0.455	0.70 (0.51-0.96)	**0.028**
rs4755391	CD44	0.23	dom	0.63 (0.42-0.94)	**0.02**	0.97 (0.64-1.47)	0.886	0.78 (0.58-1.04)	0.086
rs951193	IL1R1	0.05	dom	2.03 (1.09-3.75)	**0.02**	1.06 (0.54-2.06)	0.872	1.51 (0.96-2.37)	0.077
rs5743700	TLR2	0.06	dom	0.39 (0.17-0.91)	**0.03**	1.05 (0.57-1.95)	0.866	0.74 (0.45-1.22)	0.240

^*^Adjusted by age, gender, smoking status, carcinoma *in situ*, tumor size, tumor grade, and treatment.

BCG: Bacillus Calmette–Guérin immunotherapy; MAF: minor allele frequency; MOI: model of inheritance; rec: recessive; dom: dominant; HR: hazard ratio; CI: confidence interval. Bold font denotes significant *P*-values.

### Associations between significantly opposing inflammatory genetic variants and progression

In the discovery phase, we identified 15 significant opposing SNPs according to BCG therapy but not significant in the TUR only group (Table 4). The most significant SNP was rs1800686 in the gene CD40 which conferred a 3.8-fold (95% CI, 1.55-9.37) increased risk of progression among BCG treated patients compared to those with TUR only treatment. To internally validate these results, we again performed random bootstrap resampling analysis. In BCG-treated patients, one SNP (rs7089861 in *IL2RA*) had highly consistent results, with bootstrap *P*-values <0.05 for greater than 90% of samplings.

**Table 4:** Significantly opposing inflammatory gene variants associated with progression in NMIBC patients in the discovery cohort who received BCG and TUR only treatment

SNP	Gene	MAF	MOI	TUR only				BCG			
				# of events	# of no events	HR (95% CI)^*^	*P*-value	# of events	# of no events	HR (95% CI)^*^	*P*-value
rs1800686^#^	CD40	0.24	REC	9\10\0	74\46\5	NA	NA	24\26\7	83\59\6	3.81 (1.55-9.37	<0.01
rs2071081	CD4	0.21	REC	10\9\0	81\39\5	NA	NA	33\15\9	93\49\6	3.19 (1.36-7.47)	0.01
rs7089861	IL2RA	0.28	REC	13\6\0	66\50\9	NA	NA	28\21\8	76\60\11	3.16 (1.38-7.22)	0.01
rs1210225	CD34	0.21	DOM	12\7\0	74\47\4	0.92 (0.29-2.94)	0.89	34\21\2	96\46\6	2.11 (1.16-3.83)	0.01
rs709592^#^	CSF3	0.37	REC	10\5\4	50\59\16	0.91 (0.18-4.70)	0.91	21\21\15	61\69\16	2.24 (1.14-4.40)	0.02
rs752118^#^	CD40	0.26	REC	9\10\0	73\47\5	NA	NA	24\25\8	83\59\6	2.88 (1.22-6.80)	0.02
rs12309^#^	CSF3	0.41	DOM	6\11\2	40\64\21	1.13 (0.35-3.67)	0.83	31\13\13	48\74\26	0.52 (0.29-0.93)	0.03
rs2302777	CSF3	0.41	DOM	6\11\2	39\65\21	1.11 (0.34-3.60)	0.86	31\13\13	48\75\25	0.52 (0.29-0.93)	0.03
rs2227315^#^	CSF3	0.36	REC	10\5\4	50\58\15	0.92 (0.18-4.74)	0.92	21\20\14	60\66\16	2.15 (1.07-4.32)	0.03
rs12722588^#^	IL2RA	0.17	DOM	15\4\0	83\40\2	0.95 (0.29-3.07)	0.93	37\18\2	103\43\2	1.92 (1.04-3.57)	0.04
rs2302776^#^	CSF3	0.49	DOM	2\13\4	33\58\34	1.84 (0.39-8.64)	0.44	26\18\13	39\69\40	0.55 (0.31-0.98)	0.04
rs709591^#^	CSF3	0.37	REC	10\5\4	50\59\16	0.91 (0.18-4.70)	0.91	21\21\15	61\69\17	2.05 (1.05-4.00)	0.04
rs7093069^#^	IL2RA	0.17	DOM	15\4\0	83\40\2	0.95 (0.29-3.07)	0.93	37\18\2	103\43\2	1.92 (1.04-3.57)	0.04
rs4794823^#^	CSF3	0.37	REC	10\5\4	51\57\16	0.93 (0.18-4.80)	0.93	21\21\15	61\70\17	1.96 (1.00-3.84)	0.05
rs4795418^#^	CSF3	0.37	REC	10\5\4	51\58\16	0.91 (0.18-4.70)	0.91	21\21\15	61\70\17	1.96 (1.00-3.84)	0.05

*Adjusted by age, gender, smoking status, carcinoma *in situ*, tumor size, tumor grade, and treatment.

^#^SNPs with high linkage disequilibrium (LD; r^2^>0.8): rs1800686 and rs752118 in LD; rs12309 and rs2302777 in LD; rs709592 and rs709591 in LD; rs709592 and rs4795418 in LD; rs709592 and rs2227315 in LD; rs709592 and rs4794823 in LD; rs709591 and rs4795418 in LD rs709591 and rs2227315 in LD; rs709591 and rs4794823 in LD; rs4795418 and rs2227315 in LD; rs4795418 and rs4794823 in LD; rs2227315 and rs4798423 in LD; rs12722588 and rs7093069 in LD.

TUR: transurethral resection; BCG: Bacillus Calmette–Guérin immunotherapy; MAF: minor allele frequency; MOI: model of inheritance; Rec: recessive; dom: dominant; HR: hazard ratio; CI: confidence interval.

Among the 15 SNPs identified in the discovery phase, 3 SNPs were selected for further genotyping in the validation phase (Table 5). One SNP, rs7089861 in *IL2RA*, showed the same trend of effects between discovery and validation populations and reached statistical significance in the combined meta-analysis. In the discovery (HR =3.15, 95% Confidence Interval [CI]: 1.38-7.22, *P*<0.01), validation (HR=3.84, 95% CI: 1.64-9.0, *P*=0.002) and meta-analysis (HR=3.47, 95% CI: 1.92-6.28, *P*<0.001), rs7089861 was the only SNP significantly associated with risk of progression in the BCG treated group, respectively. Both rs1800686 and rs2071081 have probable association since HRs were of the same trend, but *P*-values were not significant during the validation phase. The meta-analysis was significant at *P*=0.002.

**Table 5:** Results for the significantly opposing inflammatory gene variants in NMIBC patients with BCG treatment associated with progression in the discovery, validation and meta-analysis

*Progression*	Gene	MAF	MOI	Discovery		Validation		Meta-analysis	
SNP				HR (95% CI)^*^	*P*-value	HR (95% CI)^*^	*P*-value	HR (95% CI)	*P*-value
rs1800686	CD40	0.24	rec	3.81 (1.55-9.37)	**<0.001**	1.91 (0.72-5.03)	0.191	2.77 (1.43-5.36)	**0.002**
rs2071081	CD4	0.21	rec	3.19 (1.36-7.47)	**0.010**	1.24 (0.43-3.53)	0.693	2.19 (1.13-4.26)	**0.002**
rs7089861	IL2RA	0.28	rec	3.15 (1.38-7.22)	**0.006**	3.84 (1.64-9.0)	**0.002**	3.47 (1.92-6.28)	**<0.001**

^*^Adjusted by age, gender, smoking status, carcinoma *in situ*, tumor size, tumor grade, and treatment.

BCG: Bacillus Calmette–Guérin immunotherapy; MAF: minor allele frequency; MOI: model of inheritance; rec: recessive; dom: dominant; HR: hazard ratio; CI: confidence interval. Bold font denotes significant *P*-values.

**Figure 1:**
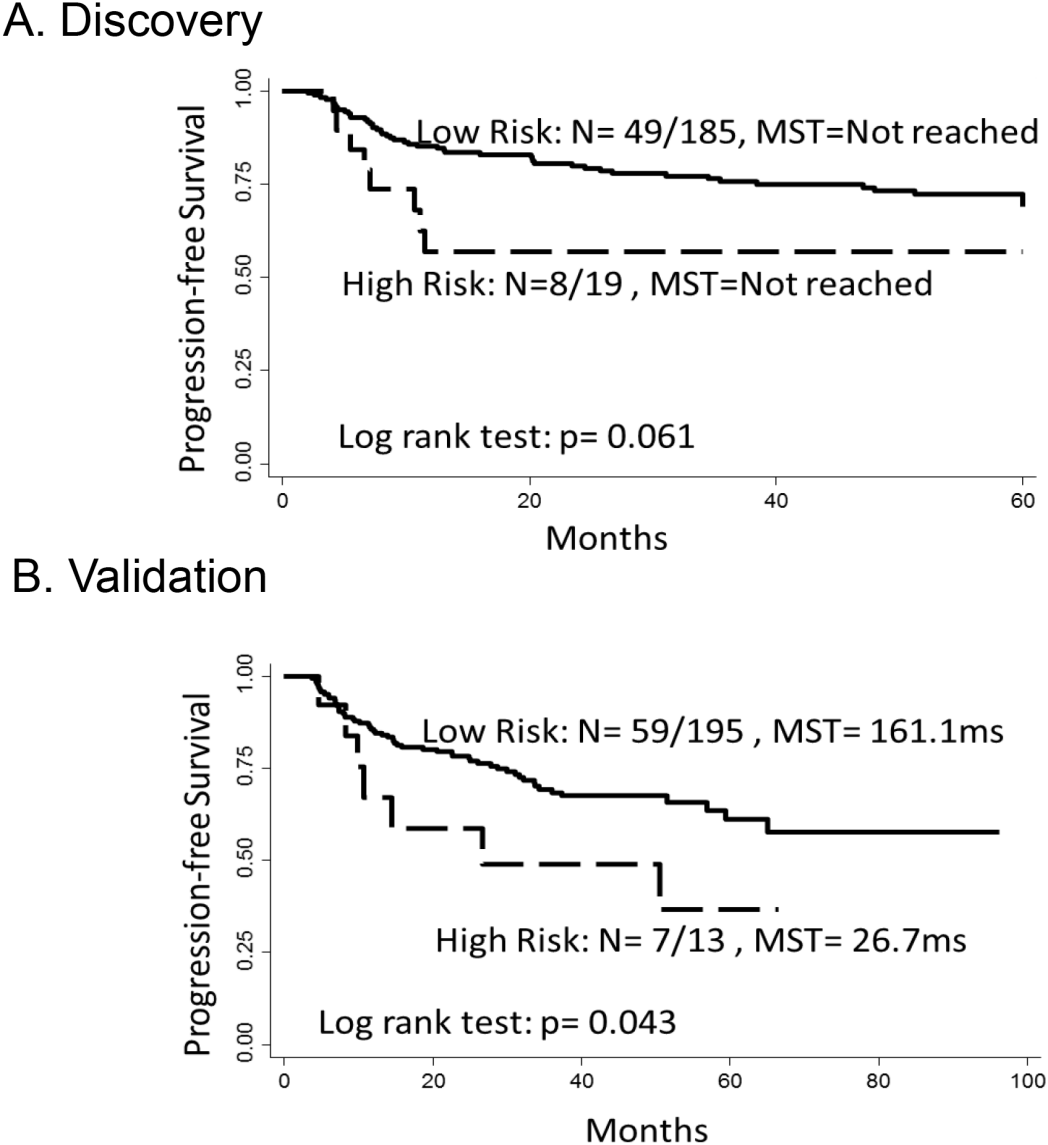
Kaplan–Meier estimates of progression-free survival in NMIBC patients Patients were grouped by the two risk groups as categorized by the presence or absence of the unfavorable rs7089861 genotype (homozygous minor alleles) in the inflammatory gene pathway. Number of events divided by total number (N) and progression-free median survival times (MST) in months were provided accordingly. **(A)** Kaplan–Meier estimates of progression-free survival in the BCG-treated group from the discovery cohort. **(B)** Kaplan–Meir estimates of progression-free survival in the BCG-treated group from the validation cohort.

Kaplan–Meier survival analysis also showed significant to borderline significant difference in progression free time by SNP rs7089861 genotype in the discovery and validation phases in patients who underwent BCG therapy (Figure 1). There was shorter median survival time (MST) with the presence of unfavorable rs7089861 genotype (homozygous minor alleles) compared to the low risk genotypes (homozygous and heterozygous wildtype alleles) (log rank *P*-values were 0.06 in the discovery phase and 0.04 in the validation phase).

### Functional characterization

We performed functional characterization of the validated SNP rs7089861 and two SNPs (rs1800686 and rs2071081) showing probable association (Supplementary Table 3). There are no additional variants in high linkage disequilibrium (LD, r^2^>0.8) with rs7089861 and rs2071081, while the region covering rs1800686 and its correlated variants (r^2^>0.8) spans 27.5 kb. All three SNPs exhibited significant effect on promoter, enhancer, DNAse hypersensitivity and regulatory motif analysis in multiple tissues, while rs1800686 and rs2071081 also showed significant effect on protein binding analysis. Furthermore, we observed strong eQTL effect on regulatory gene function for rs1800686 on expression of *CD40* and *PLTP* and rs2071081 on expression of *CD4*, *GPR162*, and *LEPREL2* in multiple tissues (Supplementary Table 4).

## DISCUSSION

We evaluated the association of variants within the inflammation pathway gene panel with recurrence and progression in patients who received BCG immunotherapy. Validation confirmed variant allele, rs7089861, was associated with risk of progression in the BCG treated group.

Of the 15 significant opposing SNPs for progression (i.e. significant for BCG therapy but not for TUR only group), we found one SNP, rs7089861, which was validated for progression in the BCG treated patients. In addition, a recent genome-wide association study showed that the association of rs7089861 with multiple sclerosis reached genome-wide significant level [10]. This SNP is in chromosome 10p15.1 and 6 kb from interleukin 2 (IL2) receptor subunit alpha (*IL2RA*) gene*.* Genomic region covering *IL2RA* is known to harbor the susceptibility loci for multiple sclerosis and type 1 diabetes [11]. Although we did not find apparent association of rs7089861 with *IL2RA* gene expression by eQTL analysis, rs7089861 showed significant functional potential through altered motifs on promoter and enhancer histone marks, DNAse hypersensitivity analysis, and the regulatory motif analysis (altered 6 motifs). *IL2RA* encodes IL-2Rα receptor protein which could form the high-affinity IL-2 receptor together with IL-2Rβ and IL-2Rγ. A soluble form of IL-2Rα could be detected in serum and altered expression level of soluble IL-2Rα have been shown to be associated with outcomes of various cancer types [12]. The IL-2/IL-2 receptor (IL-2R) pathway is critical for promoting the growth of T cell in response to antigen encounter. Further functional characterizations of IL-2Rα are needed to determine the effect of IL-2Rα on BCG response.

The most significant SNP in the discovery data was rs1800686 in the gene CD40. Although rs1800686 was not significant in the validation data, the hazard ratio in the validation was in the same direction, and the *P*-value for the meta-analysis was significant at 0.002. This SNP also showed eQTL effect with CD40 expression in skin, transformed fibroblasts, adipose, adrenal gland, adipocytes and blood samples. This gene is a member of the TNF-receptor superfamily. The encoded protein is a receptor on antigen-presenting cells of the immune system and is essential for mediating a broad variety of immune and inflammatory responses including T cell-dependent immunoglobulin class switching, memory B cell development, and germinal center formation [13]. AT-hook transcription factor AKNA is reported to coordinately regulate the expression of this receptor and its ligand, which may be important for homotypic cell interactions. Adaptor protein TNFR2 interacts with this receptor and serves as a mediator of the signal transduction [14, 15]. The interaction of this receptor and its ligand is found to be necessary for amyloid-beta-induced microglial activation, and thus is thought to be an early event in Alzheimer disease pathogenesis [16]. Mutations affecting this gene are the cause of autosomal recessive hyper-IgM immunodeficiency type 3 (HIGM3). Multiple alternatively spliced transcript variants of this gene encoding distinct isoforms have been reported [17]. The precise interplay of CD40 as it relates to NMIBC and BCG susceptibility remains to be elucidated.

Our findings must be interpreted in the context of the study design. We identified several polymorphisms associated with recurrence and progression among patients with NMIBC who received BCG immunotherapy. Since many of these inflammatory pathway SNPs are also important in immune responses, further research determining the exact interaction and potential for altered host immune response are needed to elucidate our findings. Although we considered adjustment for multiple testing, performed bootstrap sampling to internally validate these associations as well as replicated our findings in a separate group of patients, independent external studies are needed to confirm these results. Although there was no statistical difference between the discovery and validation sets for majority of the variables, age, carcinoma *in situ*, focality, and frequency of recurrence did differ between the two groups, which may lessen the likelihood of replication for some SNPs. Missing information for some clinical variables, such as tumor size and focality, may affect the final analysis in terms of adjustment for potential covariates. Additionally, we did not record specific number of maintenance courses, and we cannot comment on the SNP associations identified in the present study according to number of maintenance courses. Despite being adequately powered for overall analysis, our sample size may not be large enough to detect weaker associations and may be limited for some stratified analyses. Further validation of our findings and functional studies to identify biological basis for the observed associations are warranted.

In summary, we identified a set of SNPs within the inflammatory gene pathway associated with NMIBC outcome. One variant, rs7089861, was validated for progression and several SNPs with probable associations among patients who underwent BCG immunotherapy. Once replicated in independent studies, the identified loci may contribute to risk stratification protocol to identify patients most likely to benefit from BCG versus upfront radical cystectomy. Significant loci may point to potential therapeutic targets in the inflammation pathway to reduce the risk of NMIBC recurrence and progression.

## MATERIALS AND METHODS

### Patient population and data collection

Discovery population: Bladder cancer patients were recruited from the University of Texas MD Anderson Cancer Center and Baylor College of Medicine through a daily review of computerized appointment schedules as a part of an ongoing bladder cancer case–control study since 1995 [18]. To be eligible for this study, patients had to be histologically confirmed non-variant urothelial NMIBC and previously untreated with chemotherapy or radiation. In total, 349 NMIBC patients were selected for inclusion in the discovery. The study population has been described in detail previously [19]. There were no age, gender, ethnicity and cancer stage restrictions on recruitment.

Validation population: The validation patient population consisted of 322 histologically confirmed NMIBC cases similarly recruited from MD Anderson Cancer Center as the discovery group without any restrictions based on demographic or clinical criteria as part of an ongoing bladder cancer study. All patients were histologically confirmed NMIBC cases without prior treatment.

Patient demographic variables, tobacco and alcohol use history, family history of cancer and medical history were ascertained. Clinical information was abstracted from the medical records, including clinical stage, grade, pathological stage, histology, treatment, recurrence and progression. All patients provided written informed consent, and the study protocol has been approved by the institutional review board of MD Anderson Cancer Center and Baylor College of Medicine.

### Genotyping

Genomic DNA was isolated from peripheral blood using the QIAamp DNA blood Maxi Kit (QIAGEN, Valencia, CA) according to the manufacturer’s protocol as previously reported [18]. We combined literature exploration and database mining to select candidate genes in the inflammatory gene pathway [20]. A total of 372 SNPs in 27 selected target genes in the inflammation gene pathway were selected for genotyping and passed the quality control checking. The genotyping of the SNPs was done using Illumina’s iSelect custom SNP array platform together with other cancer-related pathway SNPs according to the manufacturer’s Infinium II assay protocol (Illumina, San Diego, CA). All of the patients’ genotypes were called and exported using BeadStudio software (Illumina). The average call rate for the SNP array was 99.7%. Among the significant SNPs associated with NMIBC outcome, 8 SNPs (5 for recurrence and 3 for progression) were selected for further validation based on their consistent data with previously unpublished data. Validation genotyping of 8 candidate SNPs was performed using Taqman (Applied Biosystems). Functional characterization of SNPs were evaluated using HaploReg [21]. Expression quantitative trait loci (eQTL) analysis was performed using various annotation tools including SNIPA [22], GTEx [23], and HaploReg [21].

### Statistical analysis

The primary endpoint events of this analysis were recurrence and progression, which was calculated from the date of diagnosis to the date of endpoint events, death or last follow-up, whichever came first. Tumor recurrence was defined as a newly detected bladder tumor following a negative follow-up cystoscopy. Tumor progression was defined as the transition from non-muscle invasive to invasive or metastatic disease. Patients who died or lost to follow-up before the endpoint events were censured. Comparisons of patient characteristics were analyzed using the Student *t*, Mann–Whitney, Chi-square or Fisher exact test, as appropriate. For smoking history patients who had never smoked or had smoked fewer than 100 cigarettes in a lifetime are considered never smokers while those who had smoked at least 100 cigarettes in a lifetime are considered ever smokers. The hazard ratio (HR) and the corresponding 95% confidence interval (CI) for the endpoints of interest were estimated by applying the multivariate Cox proportional hazards regression model while adjusting for patient age at diagnosis, gender, smoking status, stage, tumor grade and treatment.

Since several SNPs were tested in the analysis, the Q value, a measure of significance in terms of the false discovery rate, was used to adjust the significance level for multiple testing as previously reported [18, 24]. We calculated Q value by the Q value package implemented in the R software. In addition, we applied a bootstrap resampling method to internally validate the results. We generated 100 bootstrapped samples. Each time, a bootstrap sample was drawn from the original dataset and the *P* value was obtained for each SNP among the dominant, recessive and additive models. For each SNP dominant, recessive and additive models were analyzed, and only the best-fitting model was reported. Meta-analysis was performed to summarize the effects from discovery and validation populations. All statistical analyses were two sided. Kaplan–Meier curves were plotted for each genotype or unfavorable group, and log rank tests were applied to compare the difference between the event free survival time of each genotype or unfavorable group. Statistical analysis was done with Stata^®^, version 10 (College Station, TX, USA).

## SUPPLEMENTARY MATERIALS TABLES







## References

[1] Siegel RL, Miller KD, Jemal A (2016). Cancer statistics, 2016. CA Cancer J Clin.

[2] Zhao W, Schorey JS, Groger R, Allen PM, Brown EJ, Ratliff TL (1999). Characterization of the fibronectin binding motif for a unique mycobacterial fibronectin attachment protein, FAP. J Biol Chem.

[3] Ayari C, LaRue H, Hovington H, Caron A, Bergeron A, Têtu B, Fradet V, Fradet Y (2013). High level of mature tumor-infiltrating dendritic cells predicts progression to muscle invasion in bladder cancer. Hum Pathol.

[4] Kresowik TP, Griffith TS (2009). Bacillus Calmette-Guerin immunotherapy for urothelial carcinoma of the bladder. Immunotherapy.

[5] Hall MC, Chang SS, Dalbagni G, Pruthi RS, Seigne JD, Skinner EC, Wolf JS, Schellhammer PF (2007). Guideline for the management of nonmuscle invasive bladder cancer (stages Ta, T1, and Tis): 2007 update. J Urol.

[6] Redelman-Sidi G, Glickman MS, Bochner BH (2014). The mechanism of action of BCG therapy for bladder cancer—a current perspective. Nat Rev Urol.

[7] Pu X, Wang L, Chang JY, Hildebrandt MA, Ye Y, Lu C, Skinner HD, Niu N, Jenkins GD, Komaki R, Minna JD, Roth JA, Weinshilboum RM, Wu X (2014). Inflammation-related genetic variants predict toxicity following definitive radiotherapy for lung cancer. Clin Pharmacol Ther.

[8] Hildebrandt MA, Komaki R, Liao Z, Gu J, Chang JY, Ye Y, Lu C, Stewart DJ, Minna JD, Roth JA, Lippman SM, Cox JD, Hong WK (2010). Genetic variants in inflammation-related genes are associated with radiation-induced toxicity following treatment for non-small cell lung cancer. PLoS One.

[9] Guerra JL, Gomez D, Wei Q, Liu Z, Wang LE, Yuan X, Zhuang Y, Komaki R, Liao Z (2012). Association between single nucleotide polymorphisms of the transforming growth factor β1 gene and the risk of severe radiation esophagitis in patients with lung cancer. Radiother Oncol.

[10] Patsopoulos NA, Esposito F, Reischl J, Lehr S, Bauer D, Heubach J, Sandbrink R, Pohl C, Edan G, Kappos L, Miller D, Montalbán J, Polman CH, International Multiple Sclerosis Genetics Consortium (2011). Genome-wide meta-analysis identifies novel multiple sclerosis susceptibility loci. Ann Neurol.

[11] Maier LM, Lowe CE, Cooper J, Downes K, Anderson DE, Severson C, Clark PM, Healy B, Walker N, Aubin C, Oksenberg JR, Hauser SL, Compston A, International Multiple Sclerosis Genetics Consortium (2009). IL2RA genetic heterogeneity in multiple sclerosis and type 1 diabetes susceptibility and soluble interleukin-2 receptor production. PLoS Genet.

[12] Yang ZZ, Grote DM, Ziesmer SC, Manske MK, Witzig TE, Novak AJ, Ansell SM (2011). Soluble IL-2Rα facilitates IL-2-mediated immune responses and predicts reduced survival in follicular B-cell non-Hodgkin lymphoma. Blood.

[13] Flavell RA (2002). The relationship of inflammation and initiation of autoimmune disease: role of TNF super family members. Curr Top Microbiol Immunol.

[14] Ihnatko R, Kubes M (2007). TNF signaling: early events and phosphorylation. Gen Physiol Biophys.

[15] Li X, Yang Y, Ashwell JD (2002). TNF-RII and c-IAP1 mediate ubiquitination and degradation of TRAF2. Nature.

[16] Jiang H, He P, Xie J, Staufenbiel M, Li R, Shen Y (2014). Genetic deletion of TNFRII gene enhances the Alzheimer-like pathology in an APP transgenic mouse model via reduction of phosphorylated IκBα. Hum Mol Genet.

[17] Cosman D (1994). A family of ligands for the TNF receptor superfamily. Stem Cells.

[18] Chen M, Gu J, Delclos GL, Killary AM, Fan Z, Hildebrandt MA, Chamberlain RM, Grossman HB, Dinney CP, Wu X (2010). Genetic variations of the PI3K-AKT-mTOR pathway and clinical outcome in muscle invasive and metastatic bladder cancer patients. Carcinogenesis.

[19] Wei H, Kamat A, Chen M, Ke HL, Chang DW, Yin J, Grossman HB, Dinney CP, Wu X (2012). Association of polymorphisms in oxidative stress genes with clinical outcomes for bladder cancer treated with Bacillus Calmette-Guérin. PLoS One.

[20] Yang H, Gu J, Lin X, Grossman HB, Ye Y, Dinney CP, Wu X (2008). Profiling of genetic variations in inflammation pathway genes in relation to bladder cancer predisposition. Clin Cancer Res.

[21] Ward LD, Kellis M (2012). HaploReg: a resource for exploring chromatin states, conservation, and regulatory motif alterations within sets of genetically linked variants. Nucleic Acids Res.

[22] Arnold M, Raffler J, Pfeufer A, Suhre K, Kastenmüller G (2015). SNiPA: an interactive, genetic variant-centered annotation browser. Bioinformatics.

[23] Carithers LJ, Ardlie K, Barcus M, Branton PA, Britton A, Buia SA, Compton CC, DeLuca DS, Peter-Demchok J, Gelfand ET, Guan P, Korzeniewski GE, Lockhart NC, GTEx Consortium (2015). A Novel Approach to High-Quality Postmortem Tissue Procurement: The GTEx Project. Biopreserv Biobank.

[24] Storey JD, Tibshirani R (2003). Statistical significance for genomewide studies. Proc Natl Acad Sci USA.

