# Supplementary material for: Genetic variants in the inflammation pathway as predictors of recurrence and progression in non-muscle invasive bladder cancer treated with Bacillus Calmette–Guérin
Source: Oncotarget. 2017 Sep 23;8(51):88782–91. doi: 10.18632/oncotarget.21222 (PMC5687645; doi:10.18632/oncotarget.21222)
Supplement: Supplementary file 1 [file oncotarget-08-88782-s001.pdf]

Genetic variants in the inflammation pathway as predictors of recurrence and progression in non-muscle invasive bladder cancer treated with Bacillus Calmette–Guérin

SUPPLEMENTARY MATERIALS

Supplementary Table 1: Demographics of the study populations for discovery and validation groups

Variables*	Discovery (n=144)	Validation (n=113)	P-value
Age (y), Mean (SD) y	63.1 (11.3)	65.6 (10.8)	$4.68x10^{-3}$
Sex, No (%)			
Male	281 (80.5)	273 (84.8)	
Female	68 (19.5)	49 (15.2)	0.146
Smoking status, No (%)			
Never	100 (28.7)	93 (29.1)	
Former	174 (49.9)	171 (53.4)	
Current	75 (21.5)	56 (17.5)	0.410
Carcinoma in situ, No (%)			
Yes	108 (32.0)	114 (41.6)	
No	229 (68.0)	160 (58.4)	0.015
Tumor size, No (%)			
1-2cm	46 (35.1)	48 (36.1)	
2-5cm	65 (49.6)	65 (48.9)	
>5cm	20 (15.3)	20 (15.0)	0.986
Stage			
Tis	161 (46.4)	138 (44.5)	
Ta	20 (5.8)	16 (5.2)	
T1	166 (47.8)	156 (50.3)	0.802
Grade			
G1	12 (3.6)	11 (3.6)	
G2	123 (36.8)	86 (28.0)	
G3	199 (59.6)	210 (68.4)	0.056
Focality			
1	134 (56.54)	92 (44.4)	
2	24 (10.1)	37 (17.9)	
Multiple	79 (33.3)	78 (37.7)	0.014
Treatment			
iBCG	144 (41.3)	113 (35.1)	
iBCG+mBCG	205 (58.7)	209 (64.9)	0.101
Recurrence			
No	127 (36.4)	65 (57.5)	
Yes	222 (63.6)	48 (42.5)	$2.81x10^{-9}$
Progression			
No	273 (78.2)	231 (71.7)	
Yes	76 (21.8)	91 (28.3)	0.052

SD: standard deviation; BCG: bacillus Calmette-Guerin immunotherapy; iBCG: induction BCG; mBCG: maintenance BCG. Bold font denotes significant P values.

Supplementary Table 2: Selected genes and SNPs in the inflammation pathway

See Supplementary File 1

Supplementary Table 3: HaploReg analysis of rs7089861, rs2071081, rs1800686 and their correlated variants (r^2>0.80)

See Supplementary File 2

Supplementary Table 4: Expression quantitative trait locus (eQTL) analysis with direct effect on regulation of gene function

Gene Symbol	P-Value	Tissue	Source
**rs1800686**			
CD40	5.10E-09	Skin - Sun Exposed (Lower leg)	GTEx
CD40	1.00E-06	Cells - Transformed fibroblasts	GTEx
CD40	1.00E-05	Adipose - Subcutaneous	GTEx
CD40	1.50E-05	Adrenal Gland	GTEx
CD40	5.71E-05	Whole Blood	Westra et al, Nat Genet. 2013
PLTP	2.95E-06	Blood	MuTHER consortium
CD40	4.15E-05	Adipocyte	MuTHER consortium
**rs2071081**			
CD4	3.91E-07	Whole Blood	Westra et al, Nat Genet. 2013
GPR162	2.26E-26	Whole Blood	Westra et al, Nat Genet. 2013
CD4	1.10E-03	Blood	Fehrmann et al, PLoS Genet. 2011
GPR162	1.70E-08	Blood	Fehrmann et al, PLoS Genet. 2011
LEPREL2	1.37E-02	Dendritic cells after treatment with Mycobacterium tuberculosis	Barreiro et al, Proc Natl Acad Sci 2012
