# Supplementary material for: Genetic variants in the inflammation pathway as predictors of recurrence and progression in non-muscle invasive bladder cancer treated with Bacillus Calmette–Guérin
Source: Oncotarget. 2017 Sep 23;8(51):88782–91. doi: 10.18632/oncotarget.21222 (PMC5687645; doi:10.18632/oncotarget.21222)
Supplement: Supplementary file 3 [file oncotarget-08-88782-s003.pdf]

Supplemental Table 3. HaploReg analysis of rs7089861, rs2071081, rs1800686 and their correlated variants (r^2>0.80)

Chr	Position (hg38)	LD (r^2)	LD (D')	variant	Ref	Alt	AFR freq	AMR freq	ASN freq	EUR freq	Promoter histone marks	Enhancer histone marks	DNAse	Proteins bound	Motifs changed	GRASP QTL hits	eQTL hits	GENCODE genes	dbSNP func annot
**rs7089861 and variants with r^2 >= 0.8**																			
10	6068363	1	1	rs7089861	C	G	0.4	0.34	0.2	0.24	BLD	5 tissues	5 tissues		6 altered motifs			6kb 5' of IL2RA	
**rs2071081 and variants with r^2 >= 0.8**																			
12	6826466	1	1	rs2071081	A	C	0.2	0.27	0.36	0.19	SKIN	8 tissues	BLD	POL2	CDP	3 hits	2 hits	GPR162	intronic
**rs1800686 and variants with r2 >= 0.8**																			
20	46101258	1	1	rs6074019	C	T	0.1	0.15	0.37	0.27		ADRL			Gm397,Pax-4		3 hits	11kb 5' of NCOA5	
20	46102124	1	1	rs6065924	G	A	0.1	0.15	0.33	0.26			BRST,BLD	JUND	4 altered motifs	1 hit	4 hits	12kb 5' of NCOA5	
20	46104107	1	1	rs1358719	A	G	0.1	0.15	0.33	0.26					PEBP		3 hits	14kb 5' of NCOA5	
20	46104675	1	1	rs6104466	G	A	0.6	0.2	0.33	0.26					5 altered motifs		4 hits	14kb 5' of CD40	
20	46105657	1	1	rs6065925	G	A	0.1	0.15	0.33	0.26					Evi-1,GATA	2 hits	4 hits	13kb 5' of CD40	
20	46106037	1	1	rs6074020	A	T	0.1	0.15	0.33	0.26					Dmbx1,Obox6,Otx2		4 hits	12kb 5' of CD40	
20	46109011	1	1	rs6131015	G	A	0	0.14	0.33	0.26	BLD	BLD, GI, PANC	BLD,BLD		7 altered motifs		4 hits	9.3kb 5' of CD40	
20	46113362	1	1	rs1009373	G	T	0	0.14	0.33	0.26			BRST				4 hits	4.9kb 5' of CD40	
20	46114008	1	1	rs13040307	C	T	0	0.14	0.33	0.26		BRST			6 altered motifs		5 hits	4.3kb 5' of CD40	
20	46114671	1	1	rs6074024	A	G	0	0.14	0.33	0.26					BRCA1,Pou2f2	6 hits	4 hits	3.6kb 5' of CD40	
20	46117257	1	1	rs4812998	A	G	0	0.14	0.33	0.27		BLD, LIV, GI			AP-2,PRDM1,SRF		3 hits	1kb 5' of CD40	

Chr	Position (hg38)	LD (r²)	LD (D')	variant	Ref	Alt	AFR freq	AMR freq	ASN freq	EUR freq	Promoter histone marks	Enhancer histone marks	DNAse	Proteins bound	Motifs changed	GRASP QTL hits	eQTL hits	GENCODE genes	dbSNP func annot
20	46117764	1	1	rs1800686	G	A	0	0.14	0.33	0.27	21 tissues	5 tissues	14 tissues	PU1	4 altered motifs		4 hits	507bp 5' of CD40	
20	46118099	1	1	rs752118	C	T	0	0.14	0.33	0.27	19 tissues	BRN	34 tissues	5 bound proteins	SP1		4 hits	172bp 5' of CD40	
20	46119460	1	1	rs1535045	C	T	0	0.14	0.33	0.27	7 tissues	13 tissues	12 tissues	POL24H8	22 altered motifs		4 hits	CD40	intronic
20	46124597	0.9	1	rs73115010	T	C	0.1	0.15	0.34	0.26					Hlx1,Ik-2,PU.1		3 hits	CD40	intronic
20	46128768	0.8	0.9	rs3765459	G	A	0.1	0.15	0.34	0.26		6 tissues	29 tissues	4 bound proteins	ERalpha-a,Znf143		5 hits	CD40	intronic

Chr– chromosome, LD – Linkage Disequilibrium, ref – reference allele, alt – alternative allele, AFR – African American, AMR – Admixture mixed American (Mexican Ancestry from Los Angeles USA; Puerto Rican from Puerto Rica; Colombian from Medellian, Colombia; Peruvian from Lima, Peru; Gujarati Indian from Houston, Texas), ASN – Asian, EUR – European, QTL – Quantitative Trait Locus analysis, eQTL – expression QTL analysis, BLD – Blood, BRN – Brain, ADRL – Adrenal Gland, PANC – Pancreatic, LIV – Liver, BRST – Breast, GI – Gastrointestinal
